# Dysfunctional brain connectivity and cognition in late preterm infants: novel perspectives on neurodevelopment and learning

**DOI:** 10.3389/fnsyn.2026.1802655

**Published:** 2026-05-15

**Authors:** María Inés Herrera

**Affiliations:** 1 Facultad de Psicología y Psicopedagogía, Pontificia Universidad Católica Argentina, Buenos Aires, Argentina; 2 Escuela de Educación y Desarrollo Humano, Universidad de la Ciudad de Buenos Aires, Buenos Aires, Argentina

**Keywords:** cerebral connectivity, late gestation, late preterm infants, learning, neurodevelopment

## Abstract

Late preterm birth, occurred between 34 and 36 weeks of gestation, constitutes a risk factor for neurodevelopment. Despite being initially considered as a near term group of newborns, late preterm infants have shown dysfunctions in cerebral connectivity and in learning at school age. This mini-review will summarize key findings of latest studies about altered brain connectivity and cognition in this clinical population. The present work will also discuss current research gaps and brain-behavior relationships. The main objective is to contribute to optimize further investigation on the field and early intervention strategies.

## Introduction

Late preterm children, born between 34^0/7^ and 36^6/7^weeks of gestation, are known as a new risk group in Neonatology despite appearances (Sharma et al., 2021; Snyers et al., 2020). Although they are born relatively near term, this population exhibits cognitive and learning deficits from mild to moderate severity. Accounting for 70% of prematurely born children, these preterm infants constitute a unique group that increasingly challenges health and education professionals (Karnati et al., 2020).

Research using magnetic resonance imaging (MRI) and graph theoretical approaches for assessing early development of structural brain networks between 25 and 45 weeks gestational age (GA) revealed that local connectivity involving thalamus, cerebellum, superior frontal lobe, cingulate gyrus and short range cortico-cortical connections was related to the degree of prematurity (Batalle et al., 2017). Particularly, from 34 weeks GA to term, relevant developmental milestones for cerebral connectivity take place. During this last phase of the fetal period, specific circuits are selectively vulnerable to cumulative risk factors, which affect these key gestational timepoints (Kostović et al., 2021).

Therefore, the present work will revise current scientific knowledge on connectivity development in the human fetal brain throughout late gestation and summarize up-to-date evidence about dysfunctional neurodevelopment in late preterm children, considering early neurobehavioral maturation as well as cognitive performance during school trajectory. This mini-review will discuss brain-behavior relationships and offer fundamental insights toward the prevention of neurodevelopmental disorders, learning disabilities or low academic achievement.

## Brain connectivity throughout late gestation and vulnerability in late prematurity

Experts from the *Croatian Institute of Brain Research* have thoroughly studied the development of human brain connectivity from 24 weeks GA to term, by systematizing MRI and histological data (Kostović et al., 2021). As Kostović et al. (2021) emphasize, brain connectivity development is the most important neurogenetic event in terms of function. Several histogenetic processes underlie this functional connectivity and include the growth, guidance and selection of axonal pathways, together with the engagement in neuronal networks. Although these neurogenetic processes exhibit a prolonged occurrence and overlapping, differences can be detected between maturation phases. These developmental windows or “sensitive periods” are characterized by the intensity of the aforementioned processes and are defined using spatiotemporal criteria: the timing of those neurogenetic events; the spatial delineation of transient radial and tangential connectivity compartments; the maturation of white matter segments; the selection of trajectories of axonal pathways and their engagement in networks (Kostović et al., 2021).

From 24 to 27 weeks GA, the cerebral wall still shows a fetal transient pattern of compartmental organization. Anyway, sublaminar organization evidences a significant advance, as well as gliogenesis, axonal growth, neuronal maturation and migration. In addition, intensive neuronal proliferation is shown in outer subventricular zone (OSVZ), leading to waves of neurons migrating toward the cortical plate. This developmental period, which corresponds to extremely preterm (EPT) birth, could be considered as a sensory expectant or presensory connectivity phase. In the following period, from 28 to 31 weeks GA, important modifications occur in all compartments of cerebral wall. Furthermore, most afferent and efferent projection and associative pathways grow simultaneously, but gyral white matter does not develop yet, due to immature cortical convolutions. This developmental phase, which corresponds to very preterm (VPT) birth, constitutes the last period of neurogenesis for superficial cortical layers that leads to corticocortical pathways. Specifically on the preterm phase (32–36 weeks GA), the human brain shows the coexistence of transient fetal compartments and a microstructural feature revealing a pediatric-like organization (Kostović et al., 2021). Cerebral convolutions develop rapidly, and gyral white matter appears. Near term age, connectivity compartments progressively reduce their volume and complexity, evidencing a significant reorganization (Kostović et al., 2014, 2021).

As the Croatian researchers remark, the question of “when” a risk factor emerges is fundamental for investigating the etiology of neurodevelopmental dysfunctions. The histogenetic processes that take place in late gestation are different from those that occur in the first half of gestation. If birth irrumps in the last phase of gestation, the brain is selectively vulnerable to the alteration of key associative circuits, especially in the frontal, parietal and occipital cortical areas (Kostović et al., 2021).

Further investigation, involving the *Centre for the Developing Brain*, the *Centre for Neurodevelopmental Disorders* and the *Department of Neuroimaging* from the *King’s College London* in the United Kingdom, together with laboratories from Spain and Belgium, delved into spatiotemporal tissue maturation of thalamocortical pathways in the human fetal brain. As the authors indicate, connectivity development between the cortex and the thalamus is essential for the maturation of several brain functions in the second half of human gestation. Thalamocortical connections offer a crucial input for cortical areal differentiation and sensory integration circuits. This multicenter study, which was part of the *Developing Human Connectome Project*, applied high resolution *in utero* diffusion MRI from 140 fetuses, which complement histological knowledge when exploring the putative impact of preterm birth on neurodevelopment (Wilson et al., 2023). Similar to Kostović et al. (2021), Wilson et al. (2023) found that trajectories change gradually throughout GA and grouped them in three developmental windows, according to previous histological studies: early (21–25.5 weeks), mid (26–31.5 weeks) and late (32–36 weeks) prenatal periods. Results revealed an increment of tissue fraction values in the deep gray matter and the cortical plate areas with increasing GA, specially in superior brain tracts (motor, sensory and superior parietal cortex).

## Late preterm birth and dysfunctional connectivity development in brain cortex

Joseph J. Volpe, considered as the founding father of Neonatal Neurology, affirmed in 2009 that preterm birth leads to a complex amalgam of destructive and developmental disturbances. In the last years, several reports of this researcher from the *Departments of Neurology and Pediatric Newborn Medicine* at Harvard Medical School have contributed to clarify the impact of this perinatal problem on brain connectivity (Volpe, 2009). As this neurologist remarked, very preterm infants (born between 28^0/7^ and 31^6/7^weeks GA) suffer from a high incidence of intraventricular hemorrhage (IVH) and white matter injury (WMI). Instead, late preterm infants mostly exhibit gray-matter alterations, according to clinical and imaging data. Research in living preterm infants using MRI methods has shown a preponderance of disturbances in cortical development (Volpe, 2022): despite some evidence for altered microstructural growth in cerebral white matter has been reported (Kelly et al., 2016), most studies reveal a reduction in cerebral cortical volumes and an impairment in cortical gyral development (Degnan et al., 2015; Munakata et al., 2013; Walsh et al., 2014; Kim et al., 2014; Rogers et al., 2014; Wu et al., 2016; Cheong et al., 2016, 2019; Brumbaugh et al., 2016; Batalle et al., 2017). In his commentary on late preterm birth, Volpe (2022) offered an interesting overview: most of the neurodevelopmental alterations exhibited by late preterm infants occur yet in the absence of major neonatal morbidities (such as respiratory distress, hypoxemia, hypoglycemia). The neonatal neurologist explains that the particular vulnerability of the cerebral cortex to exogenous and endogenous factors related to premature extra-uterine life relies on the speed and complexity of its development in the late preterm period, which includes dendritic ramifications, synaptogenesis onset and rapid gyral growth. This gray-matter dysfunction in late preterm infants, conceptualized by Volpe (2022) as a “cerebral cortical dysmaturation,” appears as a neurobiological correlate of the cognitive and behavioral difficulties in this underappreciated clinical population.

Recent evidence using *morphologically adaptive neonatal tissue segmentation toolbox* (*MANTiS)* reinforced the idea that white matter volumes appear relatively unaffected with the increase in GA at birth, unlike deep gray matter volumes (Verschuur et al., 2025). This study, performed at the *Departments of Pediatrics* of the University of Calgary (Canada) in collaboration with several research centers from the Netherlands and the UK, explored the effect of GA at birth on brain volumes at term-equivalent age (TEA) (40–46 weeks post-menstrual age) in infants without overt brain injury born across the GA spectrum. As the authors explain, the contrast between white and gray matter vulnerability may depend on the timing of neurodevelopmental processes with respect to preterm birth (Verschuur et al., 2025): once neurogenesis is almost complete, processes like synaptic pruning and apoptosis take place for the shaping of gray matter, the refinement of neural circuits and the regulation of neuronal populations (Ortinau and Neil, 2015). Therefore, gray matter is at risk of dysmaturation in this last period of fetal development, even in the absence of brain lessions (Vasileiadis et al., 2004; Back and Miller, 2014; Back, 2017).

Another study published in the last year remarked how the brain exhibits a complex and rapid process of cortical expansion and gyrification during the final phase of gestational development (da Silva et al., 2025). These British researchers employed *anatomically constrained multimodal surface matching* (aMSM) for the quantification of cortical surface area expansion. They explored *in vivo* cortical growth trajectories using longitudinal MRI data from fetal and preterm cohorts, which was obtained as part of the *Developing Human Connectome Project*. This methodological approach allowed to compare *in utero* versus *ex-utero* cortical growth using cortical surface data from 22 to 44 weeks post-menstrual age (PMA). Results suggested there are distinct regional and temporal growth patterns during the 2nd and 3rd trimesters of healthy fetal cortical expansion. These normal trajectories are modified when the brain is developed *ex-utero* after preterm birth, thus altering cortical organization. Most interestingly, the authors emphasize that the combination of biomechanically constrained surface registration with high quality fetal and neonatal imaging may help further investigation to delve into early cortical development and putative deviations after preterm birth (da Silva et al., 2025).

## Late prematurity and neurobehavioral dysmaturation in early postnatal life

Considering cerebral dysmaturation after late preterm birth, Cheong et al. (2019) empathized the importance of neuroimaging and neurobehavioral assessment for the exploration of underlying brain structural alterations in late preterm infants. These Australian researchers, experts in *Obstetrics, Pediatrics, Neuroscience and Mental Health*, highlighted how the smaller and immature brains in this population are associated with suboptimal outcomes in neurobehavioral assessment of the newborn and with developmental delay at 2 years of age. These results underline that early neurobehavioral assessment in the postnatal period becomes an essential tool for the investigation of the neuropsychological impact of late preterm birth and for the potential prediction of subsequent alterations in child development (Cheong et al., 2019).

A previous work from this research group compared the neurobehavioral performance of infants born across different gestational age groups. Neonates were assessed with the *Neonatal Intensive Care Unit Network Neurobehavioral Scale (NNNS), the Hammersmith Neonatal Neurological Examination (HNNE) and the Prechtl’s Qualitative Assessment of General Movements (GMA).* Abnormal functioning in these scales increased with decreasing GA at birth, and was related to abnormal scores in brain MRI. In this way, the authors confirmed a continuum of neurobehavior in association with brain MRI across the GA spectrum, as well as a clear dysfunction in brain connectivity after late preterm birth (Eeles et al., 2017). Perhaps the most interesting question for neuropsychologists is how the neurobehavioral dysmaturation, already evidenced by late preterm infants in their first years of life, affects information processing and learning throughout their developmental trajectory. Recent evidence from systematic reviews and meta-analyses on cognitive and academic outcomes of late preterm birth have elucidated the main difficulties evidenced at school age. The next section will deepen this issue, commenting key contributions of these meta-analyses and of subsequent empirical studies.

## Cognitive deficits and learning difficulties in late preterm children at school

Researchers, from the *Department of Pediatrics* (Hospital of Barcelona) and the *Institute of Neuroscience* (University of Barcelona), carried out a systematic review and meta-analysis of studies involving measurements of cognitive functioning, language and academic achievement in late preterm children and respective controls at school and preschool age (Martínez-Nadal and Bosch, 2020). Following the PRISMA statement, this meta-analysis included 16 investigations that revealed suboptimal results in visuospatial perception, attention, executive functions, processing speed, short term verbal memory and literacy skills. These difficulties appeared to be subtle, conforming a complex and changing neurocognitive profile, which demands further investigation. Therefore, the authors of this systematic review remark the urgency of refining screening protocols for the early detection of neurodevelopmental risk and the mitigation of learning disabilities, especially considering the increasing incidence of late preterm birth. For this reason, they reinforce the need of applying these assessment procedures in larger samples and extending follow-up studies. In addition, Martínez-Nadal and Bosch (2020) detail the distinct assessment tools that were applied in the selected studies, standing out the value of parental questionnaires and laboratory tasks beyond standardized tests.

Another meta-analysis from 2020 delved into academic outcomes of preterm birth. Although this meta-analysis was not focused on late preterm infants specifically, it offers an interesting approach for the understanding of learning difficulties. Besides aggregate higher-order measures, a wide variety of related lower-order subskills were considered in most empirical studies and in this meta-analysis: decoding, pseudoword decoding and word identification (for reading performance); calculation, mathematical knowledge and fluency (for mathematical achievement). The systematic review selected 33 studies that compared large samples of preterm and term-born peers, aged 5–18 years and assessed with standardized tools, in the context of a cohort or a cross-sectional investigation. Statistical results revealed that preterm birth is associated with a significant deficit in reading comprehension and the resolution of applied mathematical problems, according to aggregate measures. Deficits in word identification, calculation, mathematical knowledge and fluency appeared as potential predictors of academic underachievement (McBryde et al., 2020). Applying this domain-specific approach in further research on academic performance of late preterm children may contribute significantly, particularly in this clinical subgroup whose cognitive deficits tend to be subtle (Martínez-Nadal and Bosch, 2020). Similar to Martínez-Nadal and Bosch (2020), McBryde et al. (2020) emphasize the need of designing more sensitive measures of cognitive difficulties, specially for reading and mathematical processing, beyond classical psychoeducational batteries.

McBryde et al. (2020) also offer a relevant viewpoint regarding further investigation: socioeconomic status and comorbid problems (of medical or behavioral origin) might act as mediating or moderating factors between preterm birth and academic performance. In line with this hypothesis, recent evidence reveals the potential role of early nutrition and breastfeeding on cognitive performance of late preterm infants at school age. This study, performed by Italian researchers, was focused on this population attending elementary school, specifically between 8 and 12 years of age, which were assessed with *Wechsler Intelligence Scale for Children -III (WISC-III)* by trained psychologists. A family pediatrician collected neonatal and current growth data. Results showed that late preterm children who had been fed with human milk exhibited the highest outcomes in cognitive functioning with respect to those fed with formula milk and to the mixed milk group. Therefore, the authors remark the importance of promoting breastfeeding. From this preventive perspective, they also reinforce the need of further investigation about the impact of protective factors for child health and development, specifically in late preterm children, a clinical population that is often excluded from follow-up studies (Biasini et al., 2025).

Results from a previous retrospective study performed by Japanese researchers indicated that longer breastfeeding, as well as a higher socioeconomic status, was associated with a decreased risk of neurodevelopmental impairments. Instead, low birth weight, premature rupture of membranes and smoking during pregnancy appeared as factors that increased the aforementioned risk. This investigation explored the relationship between *moderate-to-late preterm (MLPT) birth* and neurological risk in young children, compared with very preterm (VPT) and full-term (FT) birth, using large-scale population data (Lee et al., 2023). Anyway, this study referred to *moderate-to-late preterm (MLPT) children*. The nomenclature MLPT groups together two types of preterm infants. And the impact of the aforementioned factors might differ between moderately preterm (born between 32^0/7^ and 33^6/7^ GA) and late preterm (born between 34^0/7^ and 36^6/7^ GA) infants. For example, a recent multicenter study from the *Department of Women’s and Children’s Health* at the Karolinska Institutet (Sweden) reported that cognitive deficits in moderately preterm children aged 9–10 years were independent of socioeconomic status, genetics and other risk factors (Nivins et al., 2025). In addition, it is worth mentioning that the aforementioned studies were carried out in different countries, and this cultural factor may affect the results. Future studies may help to clarify the potential effects of distinct environmental and genetic factors in the respective subgroups of preterm infants.

## Discussion

The present mini-review has covered up-to-date evidence regarding neurodevelopmental dysfunction in late preterm children. Considering spatiotemporal patterns of neurogenetic events between 34 and 36 weeks GA, fundamental associative circuits are selectively vulnerable to late preterm birth, especially in frontal, parietal and occipital regions (Kostović et al., 2021). Maturation of deep gray matter and cortical plate areas might be affected, according to recent research using high resolution *in utero* diffusion MRI (Wilson et al., 2023). As several studies using neuroimaging and other methodologies have revealed, late prematurity is apparently associated with a “cortical dysmaturation” and a disruption in brain connectivity (Volpe, 2022; Verschuur et al., 2025; da Silva et al., 2025), which could be early detected using neurobehavioral testing in the newborn and developmental assessment around 2 years of age (Eeles et al., 2017; Cheong et al., 2019). According to the results from the aforementioned meta-analysis of 16 studies on cognitive functioning and academic performance in late preterm infants, this population evidences dynamic neuropsychological profiles throughout development, with subtle deficits in several cognitive processes, executive functions and literacy skills. Therefore, the refinement of screening instruments becomes mandatory, as well as the use of different types of protocols (parental questionnaires, standardized tests, laboratory tasks), and the methodological design of longer follow-up studies with larger samples of late preterm children (Martínez-Nadal and Bosch, 2020).

Taking into account the contributions of the meta-analysis on academic outcomes of preterm birth (McBryde et al., 2020), the assessment of both high- and low-order academic subskills in late preterm infants may contribute to clarify the cognitive mechanisms underlying the learning difficulties that they evidence at school and to identify early predictors of this academic underperformance. In line with this domain-specific framework, recent large-scale research on cognitive processing in preterm children delved into the distinction between *hot* (with emotional/motivational aspects) and *cool* (purely neutral/cognitive) *executive functions (EF)*, and also explored brain-behavior associations. This investigation included 3,508 children, between 9 and 10 years of age, from the *Adolescent Brain and Cognitive Development (ABCD)* study. Preterm and full term infants were followed for 4 years. Assessment included MRI scans and EF tasks. Results indicated that preterm birth may affect cool EF persistently. Instead, hot EF exhibited a catch-up pattern during early adolescence. Bivariate analyses revealed distinct bidirectional relationships in specific brain regions, suggesting disrupted cognitive-brain maturation interactions as a consequence of preterm birth. In this way, evidence challenges health and educational professionals for designing tailored interventions and long-term surveillance of preterm children (Menu et al., 2025). Furthermore, the future implementation of this domain-specific approach in EF in neuropsychological assessment of late preterm children, together with the exploration of brain-behavior relationships, may enrich investigation on cognitive and academic outcomes of late prematurity. Anyway, much more recent evidence from the same research group suggested the need of understanding the diverse spectrum of developmental trajectories beyond average outcomes, taking into account the heterogeneous profile of preterm infants (Menu et al., 2026). Further research should help to elucidate this issue, always considering the complexity of perinatal complications and its impact on neurodevelopment (Frenquelli et al., 2022), as well as the changing levels of risk for cognitive domain-specific delay over time (Stephenson et al., 2023). In this way, scientific evidence may help to determine which types of early developmental interventions are most effective in improving information processing and learning (Orton et al., 2024).

## References

[B1] Back S. A. (2017). White matter injury in the preterm infant: Pathology and mechanisms. *Acta Neuropathol.* 134 331–349. 10.1007/s00401-017-1718-6 28534077 PMC5973818

[B2] Back S. A. Miller S. P. (2014). Brain injury in premature neonates: A primary cerebral dysmaturation disorder? *Ann. Neurol.* 75 469–486. 10.1002/ana.24132 24615937 PMC5989572

[B3] Batalle D. Hughes E. J. Zhang H. Tournier J. D. Tusor N. Aljabar P. et al. (2017). Early development of structural networks and the impact of prematurity on brain connectivity. *NeuroImage* 149 379–392. 10.1016/j.neuroimage.2017.01.065 28153637 PMC5387181

[B4] Biasini A. Agostini F. Stella M. Mariani E. Malaigia L. Rizzo V. et al. (2025). Long-term neurodevelopmental outcomes of late preterm children: A pilot study on the role of early nutrition. *Nutrients* 17:3558. 10.3390/nu17223558 41305608 PMC12655716

[B5] Brumbaugh J. E. Conrad A. L. Lee J. K. DeVolder I. J. Zimmerman M. B. Magnotta V. A. et al. (2016). Altered brain function, structure, and developmental trajectory in children born late preterm. *Pediatric Res.* 80 197–203. 10.1038/pr.2016.82 27064239 PMC4990473

[B6] Cheong J. L. Y. Thompson D. K. Olsen J. E. Spittle A. J. (2019). Late preterm births: New insights from neonatal neuroimaging and neurobehavior. *Semin. Fetal Neonatal Med.* 24 60–65. 10.1016/j.siny.2018.10.003 30342897

[B7] Cheong J. L. Thompson D. K. Spittle A. J. Potter C. R. Walsh J. M. Burnett A. C. et al. (2016). Brain volumes at term-equivalent age are associated with 2-year neurodevelopment in moderate and late preterm children. *J. Pediatr.* 174 91–97. 10.1016/j.jpeds.2016.04.002 27174146

[B8] da Silva M. Liang K. Garcia K. E. Jorge Cardoso M. Robinson E. C. (2025). Differential patterns of cortical expansion in fetal and preterm brain development. *bioRxiv [Preprint]* 10.1101/2025.05.19.653958 40475433 PMC12139903

[B9] Degnan A. J. Wisnowski J. L. Choi S. Ceschin R. Bhushan C. Leahy R. M. et al. (2015). Alterations of resting state networks and structural connectivity in relation to the prefrontal and anterior cingulate cortices in late prematurity. *Neuroreport* 26 22–26. 10.1097/WNR.0000000000000296 25426826

[B10] Eeles A. L. Walsh J. M. Olsen J. E. Cuzzilla R. Thompson D. K. Anderson P. J. et al. (2017). Continuum of neurobehavior and its associations with brain MRI in infants born preterm. *BMJ Paediatr. Open* 1:e000136. 10.1136/bmjpo-2017-000136 29637152 PMC5862173

[B11] Frenquelli R. Ratcliff M. Villar, de Onis J. Fernandes M. Barros F. C. et al. (2022). Complex perinatal syndromes affecting early human growth and development: Issues to consider to understand their aetiology and postnatal effects. *Front. Neurosci.* 16:856886. 10.3389/fnins.2022.856886 35509448 PMC9058100

[B12] Karnati S. Kollikonda S. Abu-Shaweesh J. (2020). Late preterm infants - Changing trends and continuing challenges. *Int. J. Pediatr. Adolesc. Med.* 7 36–44. 10.1016/j.ijpam.2020.02.006 32373701 PMC7193066

[B13] Kelly C. E. Cheong J. L. Gabra Fam L. Leemans A. Seal M. L. Doyle L. W. et al. (2016). Moderate and late preterm infants exhibit widespread brain white matter microstructure alterations at term-equivalent age relative to term-born controls. *Brain Imaging Behav.* 10 41–49. 10.1007/s11682-015-9361-0 25739350

[B14] Kim D. J. Davis E. P. Sandman C. A. Sporns O. O’Donnell B. F. Buss C. et al. (2014). Longer gestation is associated with more efficient brain networks in preadolescent children. *NeuroImage* 100 619–627. 10.1016/j.neuroimage.2014.06.048 24983711 PMC4138264

[B15] Kostović I. Jovanov-Milošević N. Radoš M. Sedmak G. Benjak V. Kostović-Srzentić M. et al. (2014). Perinatal and early postnatal reorganization of the subplate and related cellular compartments in the human cerebral wall as revealed by histological and MRI approaches. *Brain Struct. Funct.* 219 231–253. 10.1007/s00429-012-0496-0 23250390

[B16] Kostović I. Radoš M. Kostović-Srzentić M. Krsnik Ž (2021). Fundamentals of the development of connectivity in the human fetal brain in late gestation: From 24 weeks gestational age to term. *J. Neuropathol. Exp. Neurol.* 80 393–414. 10.1093/jnen/nlab024 33823016 PMC8054138

[B17] Lee S. Han Y. Lim M. K. Lee H. J. (2023). Impact of moderate-to-late preterm birth on neurodevelopmental outcomes in young children: Results from retrospective longitudinal follow-up with nationally representative data. *PLoS One* 18:e0294435. 10.1371/journal.pone.0294435 37972123 PMC10653423

[B18] Martínez-Nadal S. Bosch L. (2020). Cognitive and learning outcomes in late preterm infants at school age: A systematic review. *Int. J. Environ. Res. Public Health* 18:74. 10.3390/ijerph18010074 33374182 PMC7795904

[B19] McBryde M. Fitzallen G. C. Liley H. G. Taylor H. G. Bora S. (2020). Academic outcomes of school-aged children born preterm: A systematic review and meta-analysis. *JAMA Netw. Open* 3:e202027. 10.1001/jamanetworkopen.2020.2027 32242904 PMC7125435

[B20] Menu I. Duffy M. Bhatia T. Trapaga S. John J. Music S. et al. (2025). Large-scale examination of hot and cool executive function in children born preterm. *Dev. Cogn. Neurosci.* 75:101593. 10.1016/j.dcn.2025.101593 40627885 PMC12272917

[B21] Menu I. Ji L. Bhatia T. Duffy M. Hendrix C. L. Thomason M. E. (2026). Beyond average outcomes: A latent profile analysis of diverse developmental trajectories in preterm and early term-born children from the adolescent brain cognitive development study. *Child Dev.* 96 36–54. 10.1111/cdev.14143 39136075 PMC11693488

[B22] Munakata S. Okada T. Okahashi A. Yoshikawa K. Usukura Y. Makimoto M. et al. (2013). Gray matter volumetric MRI differences late-preterm and term infants. *Brain Dev.* 35 10–16. 10.1016/j.braindev.2011.12.011 22285528

[B23] Nivins S. Padilla N. Kvanta H. Ådén U. (2025). Gestational age and cognitive development in childhood. *JAMA Netw. Open* 8:e254580. 10.1001/jamanetworkopen.2025.4580 40227687 PMC11997729

[B24] Ortinau C. Neil J. (2015). The neuroanatomy of prematurity: Normal brain development and the impact of preterm birth. *Clin. Anatomy* 28 168–183. 10.1002/ca.22430 25043926

[B25] Orton J. Doyle L. W. Tripathi T. Boyd R. Anderson P. J. Spittle A. (2024). Early developmental intervention programmes provided post hospital discharge to prevent motor and cognitive impairment in preterm infants. *Cochrane Database Syst. Rev.* 2:CD005495. 10.1002/14651858.CD005495.pub5 38348930 PMC10862558

[B26] Rogers C. E. Barch D. M. Sylvester C. M. Pagliaccio D. Harms M. P. Botteron K. N. et al. (2014). Altered gray matter volume and school age anxiety in children born late preterm. *J. Pediatr.* 165 928–935. 10.1016/j.jpeds.2014.06.063 25108541 PMC4252475

[B27] Sharma D. Padmavathi I. V. Tabatabaii S. A. Farahbakhsh N. (2021). Late preterm: A new high risk group in neonatology. *J. Maternal-fetal Neonatal Med.* 34 2717–2730. 10.1080/14767058.2019.1670796 31575303

[B28] Snyers D. Lefebvre C. Viellevoye R. Rigo V. (2020). La prématurité tardive : des nourrissons fragiles malgré les apparences [Late preterm : High risk newborns despite appearances]. *Revue medicale de Liege* 75 105–110. French 32030935

[B29] Stephenson N. L. Tough S. Williamson T. McDonald S. McMorrris C. Metcalfe A. (2023). Early childhood trajectories of domain-specific developmental delay and gestational age at birth: An analysis of the all our families cohort. *PLoS One* 18:e0294522. 10.1371/journal.pone.0294522 38150466 PMC10752539

[B30] Vasileiadis G. T. Gelman N. Han V. K. Williams L. A. Mann R. Bureau Y. et al. (2004). Uncomplicated intraventricular hemorrhage is followed by reduced cortical volume at near-term age. *Pediatrics* 114 e367–e372. 10.1542/peds.2004-0500 15342899

[B31] Verschuur A. S. van Wezel-Meijler G. Low S. Nijholt I. M. Metcalfe A. Skiffington J. et al. (2025). Trends in term-equivalent age brain volumes in infants born across the gestational age spectrum. *Children* 12:1026. 10.3390/children12081026 40868478 PMC12384106

[B32] Volpe J. J. (2009). Brain injury in premature infants: A complex amalgam of destructive and developmental disturbances. *Lancet Neurol.* 8 110–124. 10.1016/S1474-4422(08)70294-1 19081519 PMC2707149

[B33] Volpe J. J. (2022). Commentary - The late preterm infant: Vulnerable cerebral cortex and large burden of disability. *J. Neonatal-Perinatal Med.* 15 1–5. 10.3233/NPM-210803 34219675 PMC8842754

[B34] Walsh J. M. Doyle L. W. Anderson P. J. Lee K. J. Cheong J. L. (2014). Moderate and late preterm birth: Effect on brain size and maturation at term-equivalent age. *Radiology* 273 232–240. 10.1148/radiol.14132410 24914576

[B35] Wilson S. Pietsch M. Cordero-Grande L. Christiaens D. Uus A. Karolis V. R. et al. (2023). Spatiotemporal tissue maturation of thalamocortical pathways in the human fetal brain. *eLife* 12:e83727. 10.7554/eLife.83727 37010273 PMC10125021

[B36] Wu X. Wei L. Wang N. Hu Z. Wang L. Ma J. et al. (2016). Frequency of spontaneous BOLD signal differences between moderate and late preterm newborns and term newborns. *Neurotoxicity Res.* 30 539–551. 10.1007/s12640-016-9642-4 27316633

